# Enhanced Phosphorus Removal by Iron-Carbon in Constructed Wetlands Across Salinity Gradients: Mechanisms and Microbial Insights for Aquaculture Tailwater Treatment

**DOI:** 10.3390/biology14101459

**Published:** 2025-10-21

**Authors:** Rui Yin, Boan Chen, Xinyu He, Chen Cai, Tangfang Zhai, Haoyang Shi, Na Li, Xiaona Ma

**Affiliations:** 1 Jiangsu Key Laboratory of Marine Biotechnology, Jiangsu Ocean University, Lianyungang 222005, China; pursuit9999@163.com (R.Y.); m18153113037@163.com (B.C.); xinyu6737@163.com (X.H.); caichenninenine@163.com (C.C.); ztflec@163.com (T.Z.); 15370627319@163.com (H.S.); bey1130@163.com (N.L.); 2 Co-Innovation Center of Jiangsu Marine Bio-Industry Technology, Jiangsu Ocean University, Lianyungang 222005, China; 3 Zhejiang Yuhang Perch & Mandarin Fish Science and Technology Backyard, Hangzhou 310058, China; 4 Jiangsu Key Laboratory of Marine Bioresources and Environment, Jiangsu Ocean University, Lianyungang 222005, China; 5 College of Bio-Systems Engineering and Food Science, Zhejiang University, Hangzhou 310058, China

**Keywords:** iron-carbon constructed wetlands, salinity gradient, phosphorus removal efficiency, aquaculture tailwater, phosphorus-accumulating organisms (PAOs)

## Abstract

**Simple Summary:**

This study explored how different salinity levels affect phosphorus removal in iron-carbon constructed wetlands (IC-CWs) used for aquaculture tailwater treatment. Four systems with salinities of 0, 10, 20, and 30 were operated for 155 days. The results showed that moderate salinity (20) achieved the best performance, removing nearly 79% of total phosphorus. This efficiency was linked to stronger iron release, active microbial metabolism, and upregulation of phosphorus-related genes (such as *pstS* and *phnE*). Moderate salinity promoted beneficial bacteria like *Bacteroidota* and enzymes that enhance phosphorus cycling, while high salinity (30) inhibited microbial activity. This study also found that iron corrosion and passivation dynamics influenced phosphorus precipitation. Overall, maintaining salinity around 20 optimizes both chemical and biological phosphorus removal, offering an effective strategy for treating saline aquaculture wastewater sustainably.

**Abstract:**

Saline aquaculture tailwater challenges conventional constructed wetlands (CWs) with their limited phosphorus (P) removal capacity. To address this, iron-carbon constructed wetlands (IC-CWs) were developed and operated under four salinity gradients (0, 10, 20, and 30) for 155 days to investigate the effects of salinity on P removal and associated microbial mechanisms. The results showed that salinity critically influenced long-term P removal, with the system at salinity 20 (S20) achieving the highest total phosphorus (TP) removal efficiency (78.80 ± 6.01%). Enhanced P removal was primarily attributed to the upregulation of phosphate transport genes (*pstS*, 14.25-fold increase) and elevated activity of key enzymes (AKP and ACP) in phosphorus-accumulating organisms (PAOs). However, high salinity (30) suppressed microbial metabolic functions. Metagenomic analysis revealed that salinity stress reshaped microbial community structure, with *Bacteroidota* abundance increasing 10-fold in S20 compared to S0 (control). This phylum harbored the *phnE* gene, significantly promoting organic phosphorus mineralization. Additionally, iron release increased with rising salinity, and the relative abundance of the *phnE* gene in *Bacteroidota* was highest in the S20 group, indicating a close association between iron release and PAOs as well as organic P mineralization genes. The quadratic polynomial model revealed that iron release under high salinity followed nonlinear kinetics, with passivation layer rupture promoting iron-phosphorus precipitate desorption in later stages. These findings provide a theoretical basis for optimizing salinity parameters to enhance chemical-biological P removal synergy, offering a promising strategy for saline aquaculture wastewater treatment.

## 1. Introduction

The rapid expansion of marine aquaculture has led to increased discharge of nutrient-rich tailwater, containing high levels of organic matter, nitrogen (N), and phosphorus (P). Excessive P discharge contributes to eutrophication, promoting harmful algal blooms and hypoxia, which disrupt marine ecosystem balance [1]. Current P removal methods—physical adsorption, chemical precipitation, and biological treatment—face limitations in efficiency and cost-effectiveness [2]. Physical adsorption relies on high-surface-area materials [3], while chemical precipitation employs metal ions (e.g., Fe^3+^, Al^3+^) to form insoluble phosphates [4]. Biological removal, mediated by phosphorus-accumulating organisms (PAOs), exploits microbial uptake under aerobic-anaerobic cycling [5]. These methods often suffer from high operational costs, strong dependence on chemical additives, and risks of secondary pollution. In contrast, IC-CWs can operate stably under saline conditions, featuring low cost, low energy consumption, and strong sustainability.

Constructed wetlands (CWs) offer an eco-friendly alternative, integrating substrate filtration, plant uptake, and microbial degradation [6,7,8]. In recent years, CWs have been widely applied for the treatment of marine aquaculture tailwater. However, traditional CWs suffer from rapid substrate saturation and limited P removal efficiency. Recent studies highlight iron-based substrates as a promising solution, enhancing P removal via chemical precipitation [9]. Zero-valent iron (ZVI) generates Fe^2+^/Fe^3+^, forming stable Fe-P precipitates [10]. Coupling Fe with carbon (e.g., activated carbon) creates an iron-carbon (IC) micro-electrolysis system, in which carbon acts as the cathode material to accelerate iron corrosion and enhance the release of Fe^2+^, accelerating Fe corrosion and P adsorption [11]. Meanwhile, Iron enhances the activity of phosphorus-removing microorganisms through three mechanisms: first, it serves as a metal cofactor for key enzymes, promoting energy metabolism and polyphosphate reactions; second, it regulates cellular redox balance and increases antioxidant enzyme activity to maintain metabolic stability; and third, it acts as an electron donor under anoxic conditions, facilitating energy utilization and gene expression in denitrifying polyphosphate-accumulating organisms, thereby improving biological phosphorus removal efficiency.

Compared with alternative technologies such as zero-valent iron (ZVI)-modified wetlands, iron-manganese oxide systems, or vertical-horizontal hybrid constructed wetlands (VFCW-HFCW), IC-CWs maintain superior phosphorus removal efficiency and microbial activity under saline conditions [12]. Their low energy demand, absence of chemical additives, and long-term reusability of media highlight their novelty and sustainability for saline aquaculture tailwater treatment. Current research demonstrates that iron-carbon constructed wetlands (IC-CWs) exhibit remarkable P removal capabilities in wastewater treatment, with TP removal rates reaching 67.60–98.20% [13]. Therefore, IC-CWs utilize an internal micro-electrolysis process that couples chemical and biological reactions, enhancing Fe–P precipitation, accelerating electron transfer, and maintaining redox stability under saline conditions. Therefore, they are particularly suitable for the treatment of marine aquaculture tailwater. In addition, iron-carbon constructed wetlands (IC-CWs) exhibit a long service life and low operational and maintenance costs, allowing sustainable operation over multiple years without frequent replacement of media. Ecologically, IC-CWs promote phosphorus cycling and microbial diversity, reducing eutrophication risk in discharged water. Economically, their low energy demand and independence from external carbon sources help aquaculture enterprises lower wastewater treatment costs, supporting sustainable and green aquaculture practices.

Despite these advances, the impact of salinity on IC-CWs remains poorly understood. High salinity may (1) compete with phosphate for binding sites (e.g., Na^+^/Cl^−^ interference) and (2) inhibit functional microorganisms [14]. Paradoxically, seawater’s high ionic strength could enhance IC micro-electrolysis by promoting Fe dissolution. Additionally, Fe influences microbial P removal by modulating PAO activity—low Fe concentrations stimulate PAOs, whereas excess Fe inhibits anaerobic P release [15]. In particular, how iron valence transformation (Fe^0^–Fe^2+^–Fe^3+^) influences the activity and gene expression of phosphorus-accumulating organisms (PAOs) under salinity stress is still unclear. Given the varying salinity requirements of aquaculture species, understanding its effects on IC-CWs is critical.

Salinity in typical marine aquaculture tailwaters ranges between 15 and 35 depending on cultured species such as *Litopenaeus vannamei* and *Exopalaemon carinicauda* [14,16]. The experimental salinity levels (0, 10, 20, and 30) were therefore designed to simulate conditions from freshwater recirculation systems to high-salinity marine aquaculture environments. This study constructed four identical IC-CWs with adjusted influent salinity to systematically explore how salinity fluctuations affect phosphorus removal efficiency, the response patterns of PAOs communities under salinity stress, and the mechanistic role of iron in mediating PAO activity. This study provides preliminary insights that may support the optimization of IC-CWs for saline aquaculture wastewater treatment and improve understanding of the coupled chemical-biological mechanisms of phosphorus removal.

## 2. Material and Methods

### 2.1. System Setup

**Figure 1:**
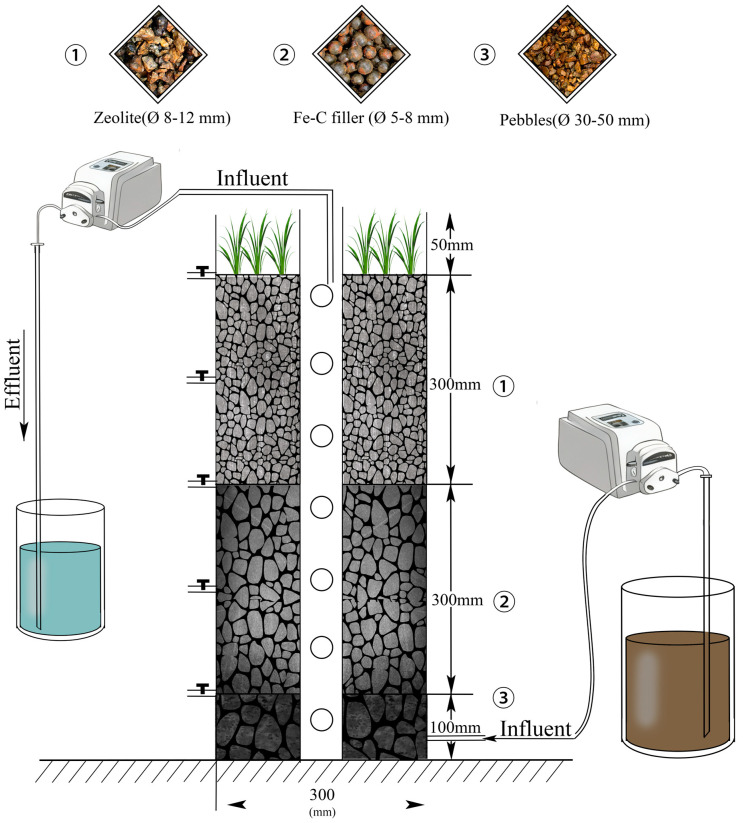
Iron-Carbon constructed wetland reactors.

Four identical upflow vertical subsurface IC-CWs (30 cm × 30 cm × 80 cm, L × W × H) were constructed (Figure 1), the reactor was designed based on an IC-CW configuration previously validated in our laboratory. The influent was supplied at a constant rate using a peristaltic pump to ensure uniform hydraulic distribution. Each filled with a 70 cm stratified substrate: 10 cm cobblestones (12–15 mm diameter) as bottom layer for drainage support; 30 cm mixed zeolite and iron-carbon substrate (8–10 mm diameter, 33% *v*/*v* iron-carbon) as middle layer for enhanced phosphorus adsorption; 30 cm fine zeolite (3–5 mm diameter) as upper layer for microbial attachment. The halophyte *Sesuvium portulacastrum* was planted at a density of 440 plants/m^2^ (12 plants/system, average fresh weight 3.50 ± 0.20 g). Four salinity treatments (0, 10, 20, and 30) of aquaculture tailwater were designed to feed into the corresponding IC-CWs, designated as S0, S10, S20, and S30, respectively [17]. Synthetic aquaculture tailwater was continuously fed into each system at 13.89 mL/min using a peristaltic pump. A perforated PVC pipe (75 cm height × 5.5 cm diameter) was installed in the center for in situ monitoring of temperature, DO, pH.

### 2.2. Experimental Design

#### 2.2.1. Operation of IC-CWs

The experiment was conducted for 155 days, from February to August. Synthetic aquaculture tailwater prepared with NH_4_Cl, NaNO_2_, NaNO_3_, C_6_H_12_O_6_, and KH_2_PO_4_ (Sinopharm Chemical Reagent Co., Ltd., Shanghai, China)was feed into the IC-CWs, containing the following parameters: chemical oxygen demand (COD) at 14.40 ± 0.85 mg L^−1^, ammonium nitrogen (NH_4_^+^-N) at 3.92 ± 0.20 mg L^−1^, nitrate nitrogen (NO_3_^−^-N) at 14.05 ± 1.12 mg L^−1^, nitrite nitrogen (NO_2_^−^-N) at 2 mg L^−1^, total nitrogen (TN) at 15.23 ± 0.59 mg L^−1^, active phosphate-phosphorus (PO_4_^3−^-P) at 4.05 ± 0.87 mg L^−1^, and total phosphorus (TP) at 4.18 ± 0.50 mg L^−1^. The salinity of aquaculture tailwater was adjusted using commercial sea salt crystals, and salinity was measured with a salinometer before each water addition to ensure that the actual salinity did not differ significantly from the preset value. The composition and nutrient levels of the synthetic aquaculture tailwater were formulated according to, representing typical conditions of marine shrimp aquaculture effluent (TN ≈ 15–20 mg L^−1^; TP ≈ 3–5 mg L^−1^) [18]. The hydraulic retention time (HRT) was set to 24 h. After the initial 10 days designated as the system acclimation phase to stabilize operational conditions, formal influent and effluent water samples were collected at three-day intervals to monitor water quality parameters. Temperature, DO, and pH were measured directly using a handheld DO meter and pH meter, PO_4_^3−^-P and TP were analyzed according to the Specifications for Oceanographic Monitoring—Part 4: Seawater Analysis (GB 17378.4-2007) [19]. The concentration of total iron (TFe) in water was quantified via the 1,10-phenanthroline spectrophotometric method (HJ/T 345-2007) [20].

#### 2.2.2. Batch Experiment

To further investigate P removal in IC-CWs treating aquaculture tailwater under varying salinity conditions, a 24 h batch experiment was conducted following the 155-day trial, aiming to enhance the resolution of salinity perturbation signatures on phosphorus migration pathways along the temporal dimension. Sampling was performed at 4 h intervals to capture the instantaneous coupling relationships among phosphorus speciation transformation, functional microbial activity, and environmental factors, thereby analyzing the dynamics of P removal processes within IC-CWs over the 24 h period.

#### 2.2.3. Validation with *Exopalaemon carinicauda* Culture Effluent

To validate the impact of salinity on phosphorus removal performance in treating *Exopalaemon carinicauda* culture effluent, tailwater samples meeting the predetermined optimal salinity conditions were collected from an aquaculture facility. Prior to the experiment commencement, key water quality parameters—including TN (16.72 ± 2.21 mg L^−1^), TP (3.23 ± 2.07 mg L^−1^), and COD (15.78 ± 2.37 mg L^−1^) were measured. The characterized tailwater was then divided into two groups: low-concentration group (TP: 4.43 ± 1.09 mg L^−1^) and high-concentration group (TP: 1.09 ± 0.53 mg L^−1^). With HRT set at 24 h, effluent was sampled every 4 h for analysis of TP, PO_4_^3−^-P concentrations.

### 2.3. Substrate Sample Collecting and Analysis

#### 2.3.1. Metagenomic Sequencing Sample Collection and Analytical Methods

Microbial community structure and the abundance of microbial genes was analyzed via metagenomic sequence. Samples for metagenomic sequence were washed with sterile physiological saline, and agitation was applied to detach microorganisms from the substrate media. The resulting suspension was filtered through a 0.22 μm membrane filter under vacuum, concentrating the microorganisms onto the membrane. The microbial-laden filters were immediately stored at −80 °C for preservation. Using extracted sample DNA as the template, bacterial V4–V5 regions of 16S rRNA were amplified with forward primer 515F (5′-GTGCCAGCMGCCGCGG-3′) and reverse primer 907R (5′-CCGTCAATTCMTTTRAGTTT-3′). Parallel sample groups were prepared and pooled to construct composite samples for metagenomic sequencing analysis. Metagenomic sequencing was performed on the Illumina NovaSeq™ X Plus platform (Illumina, San Diego, CA, USA). Metagenomic analysis was performed on the samples to obtain their microbial community and functional genes. Microbial community structure and the abundance of microbial genes was analyzed, and the effects of salinity and iron concentration on microbial phosphorus removal efficiency were investigated.

#### 2.3.2. Substrate Sample Enzyme Activity Assay Methods

Alkaline phosphatase (*AKP*) and acid phosphatase (*ACP*) activities were quantified using commercially available 48-well ELISA kits (Scistd Testing, Qingdao, China) following the manufacturer’s protocols.

### 2.4. Statical Analysis

Each salinity treatment was conducted in triplicate (*n* = 3) to ensure statistical robustness. Samples were collected from each replicate system for all analyses, and results are expressed as mean ± standard deviation (SD). All data were analyzed using SPSS 27 statistical software. One-way analysis of variance (ANOVA) was employed to examine differences among measured parameters, with statistical significance defined at *p* ≤ 0.05. Graphical representations were generated using Origin Pro 2022 and Graphpad Pism 9.5.

## 3. Results and Discussion

### 3.1. Phosphorus Removal Efficiency in IC-CWs Under Varying Salinity Conditions

#### 3.1.1. Long-Term Monitoring of Phosphorus Removal Efficiency

**Figure 2:**
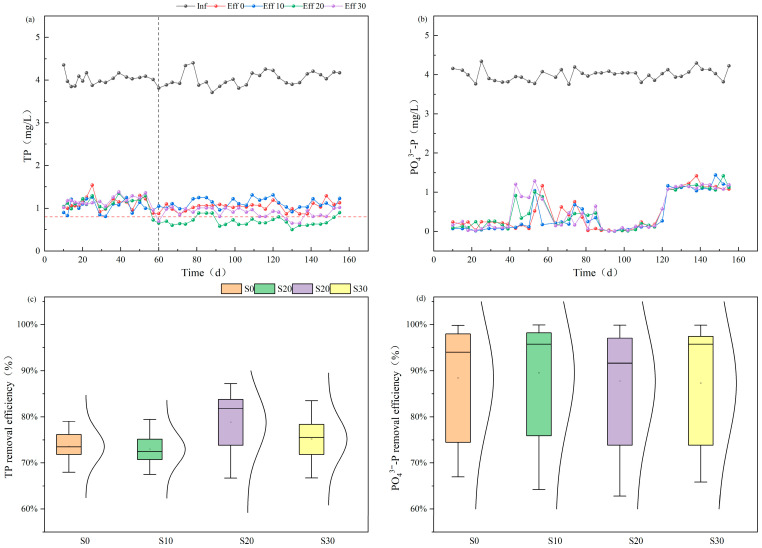
Effects of salinity on TP (**a**) and PO_4_^3−^-P (**b**) concentration and removal efficiency (**c**,**d**) in IC-CWs. Note: The red dashed line represents the Grade II discharge standard for P in aquaculture wastewater of Jiangsu Province.

A comparative analysis was conducted to investigate the effects of salinity on the removal efficiency of TP and PO_4_^3−^-P in IC-CWs (Figure 2).

For TP removal, the efficiency of the four systems showed no significant differences during the initial 60 days, with effluent TP concentration, measuring 1.10 ± 0.17 mg L^−1^ (S0), 1.04 ± 0.15 mg L^−1^ (S10), 1.09 ± 0.18 mg L^−1^ (S20), and 1.14 ± 0.16 mg L^−1^ (S30), respectively. Only partial samples met the Grade II discharge standard for aquaculture wastewater in Jiangsu Province (TP ≤ 0.8 mg L^−1^). The subsequent stabilization period (60–155 days) revealed significant performance improvements (*p* < 0.05), with average effluent concentrations decreasing to 1.04 ± 0.10 mg L^−1^ (S0), 1.12 ± 0.11 mg L^−1^ (S10), 0.69 ± 0.11 mg L^−1^ (S20), and 0.90 ± 0.12 mg L^−1^ (S30), corresponding to removal efficiencies of 74.77 ± 2.80%, 72.97 ± 2.00%, 83.22 ± 2.13%, and 78.13 ± 2.79%, respectively. Compared with conventional CWs treating saline wastewater, which typically achieve TP removal efficiencies of 40–60%, the IC-CW system in this study exhibited markedly higher efficiency (up to 83.22%), confirming the synergistic advantage of Fe–C coupling for P immobilization under saline conditions. Notably, the S20 system demonstrated optimal performance, with effluent TP concentrations significantly lower than the other three groups (*p* < 0.05), approaching Grade I standards (TP ≤ 0.5 mg L^−1^), while both S20 and S30 consistently met Grade II requirements.

For PO_4_^3−^-P removal, the results showed periodic fluctuations trend through 155-day monitoring. The effluent phosphate concentration stabilized during the initial 40 days and significantly increased (*p* < 0.05) during the period of 40–85 days, with significant differences (*p* < 0.05) observed among the four groups. From 85 to 120 days, the effluent concentration decreased significantly compared to the previous phase, returning to levels comparable to those of the first 40 days. Between 120 and 155 days, PO_4_^3−^-P concentrations rose significantly again, with no significant differences among the four groups, and the average effluent concentration stabilized at 1.15 ± 0.09 mg L^−1^.

The removal mechanism of phosphorus—including both TP and PO_4_^3−^-P—can be attributed to two main pathways: (1) physical adsorption by substrate surfaces and (2) microbial accumulation mediated by PAOs. Metagenomic sequencing revealed that in the S20 system, the relative abundance of Bacteroidota increased significantly (15.4%), accompanied by upregulation of the *phnE* and *pstS* genes, verifying the microbial mechanism of organic phosphorus mineralization that promoted P removal in this study. The results indicate that Fe^2+^ release not only facilitates chemical precipitation but also stimulates the expression of phosphatase-related genes, forming a synergistic iron-microbe pathway for phosphorus removal. However, variations in salinity not only alter the efficiency of physical adsorption but also modulate microbial activity and the regulation of functional genes, collectively shaping the overall phosphorus removal performance of IC-CWs. On one hand, excessive salinity suppresses the metabolic activity of PAOs, thereby reducing the biological removal efficiency of dissolved PO_4_^3−^-P [21]. On the other hand, elevated salinity increases electrical conductivity, which accelerates anodic reactions and enhances the electrochemical corrosion capacity of seawater [22]. The resulting iron corrosion products can precipitate with phosphorus compounds, thus contributing to the removal of TP. Although high salinity inhibits microbial activity, it enhances the coagulation and precipitation effects of Fe(III) and Fe(II) generated by the internal electrolysis system [17]. In contrast, low-salinity environments support the metabolic activity of PAOs, promoting the biological removal of PO_4_^3−^-P. Consequently, during the initial 60 days of the experiment, phosphorus removal mainly depended on substrate adsorption, as microbial communities had not yet adapted to the new environment, leading to impaired physiological function and reduced metabolic activity. After 60 days, fluctuations in effluent PO_4_^3−^-P concentrations were significantly reduced, indicating that PAOs had acclimated to the aquatic environment and started contributing to the biological removal process.

The observed increase in effluent PO_4_^3−^-P concentrations during prolonged operation may result from two mechanisms: (1) the formation of passivation layers on the surface of iron-carbon substrates. Specifically, FeOOH reacts with Fe^2+^ to form Fe_3_O_4_, and these iron oxides adhere to the sponge iron surface, decreasing its adsorption capacity for PO_4_^3−^-P [23]; (2) rising water temperatures during operation accelerate microbial metabolism and lead to the breakdown of precipitates, releasing previously adsorbed or precipitated phosphorus back into the aqueous phase. This reduces the removal efficiency of both TP and PO_4_^3−^-P. Simultaneously, the disintegration of Fe(III) and Fe(II) flocculants releases some bound iron ions, which can rebind with phosphate to form new precipitates, thereby partially restoring PO_4_^3−^-P removal and lowering its concentration in the aqueous phase. However, these interpretations remain speculative because direct evidence (e.g., SEM or XPS characterization) was not obtained in this study. Similar temperature- and passivation-related effects on phosphorus release have been reported in previous studies [24], and further material analyses are needed to confirm the occurrence and mechanism of passivation layer rupture.

#### 3.1.2. Phosphorus Removal Efficiency of Batch Experiment

**Figure 3:**
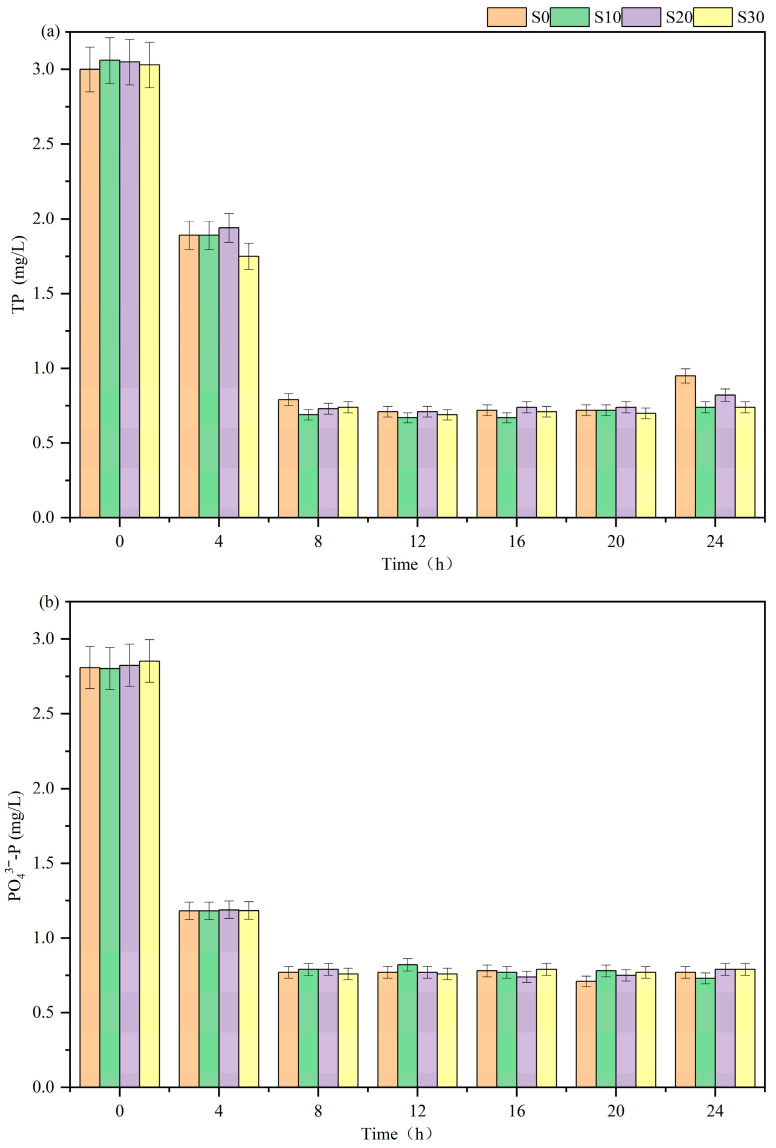
Concentration of TP (**a**) and PO_4_^3−^-P (**b**) in each system of batch experiments.

The results of 24 h batch experiment are illustrated in Figure 3. All IC-CWs maintained total phosphorus (TP) concentrations at approximately 0.8 mg L^−1^ within 8 h. Furthermore, changes in salinity exhibited no impact on phosphorus removal by IC-CWs over the 24 h period, with no significant differences observed in effluent concentrations among all systems. These findings align with the results reported by Zheng et al. [25]. A short-term alteration in salinity does not affect the phosphorus removal capacity of IC-CWs [24].

Combined with results from the 155-day continuous experiment, the impact of salinity on the P removal capacity of IC-CWs appears more pronounced over the long term and potentially exhibits periodicity. Future studies should extend experimental durations and conduct long-term monitoring across multiple operational cycles.

#### 3.1.3. Phosphorus Removal Efficiency When Treating Aquaculture Tail Water of *Exopalaemon carinicauda*

**Figure 4:**
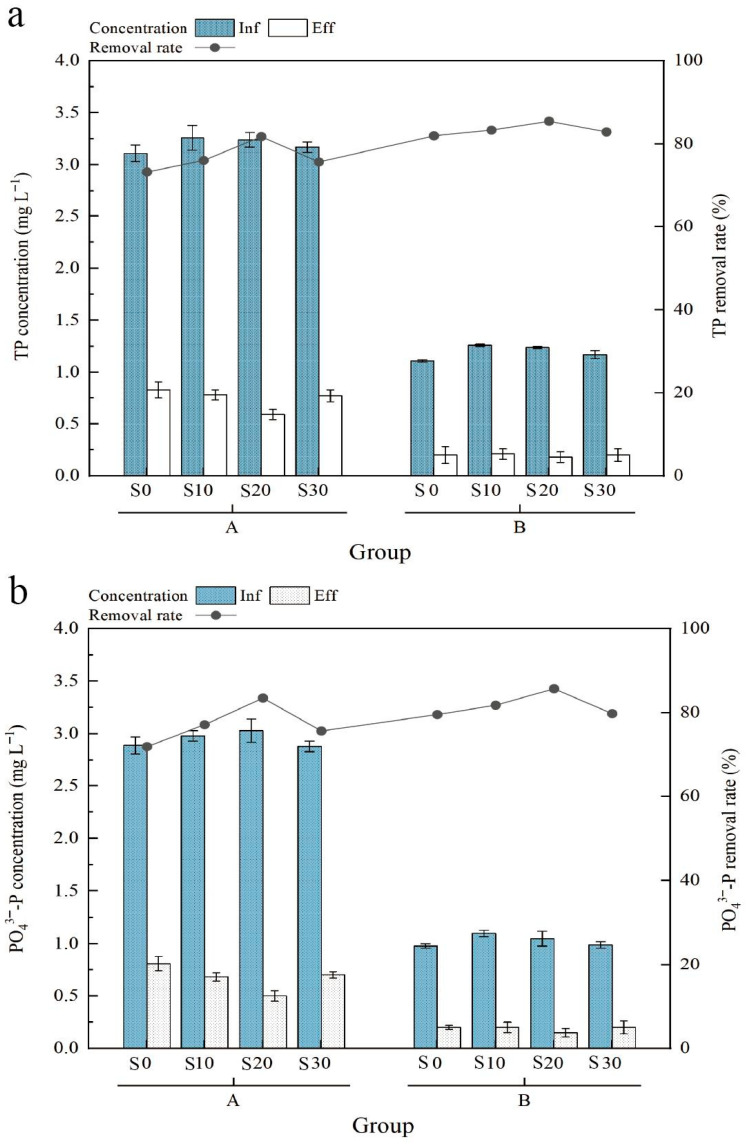
Dynamics of TP (**a**), PO_4_^3−^-P (**b**) and concentrations in aquaculture tailwater treatment group A/B.

To validate the impact of salinity on phosphorus removal efficiency of IC-CWs treating *Exopalaemon carinicauda* aquaculture tailwater at a HRT of 24 h, different influent phosphorus loading conditions were established. The results revealed that in Group A (high loading, TP: 3.20 ± 0.07 mg L^−1^), the S20 system achieved significantly higher removal efficiencies for both TP (81.79%) and PO_4_^3−^-P (83.50%) compared to other salinity treatments (S10: 76.07% and 77.18%; S30: 75.71% and 75.69%; S0: 73.31% and 71.97%; *p* < 0.01) (Figure 4). Moreover, both TP and PO_4_^3−^-P removal efficiencies increased by 5% to 8%, suggesting that lower loading mitigates substrate adsorption saturation and microbial inhibition. Experimental results validated the long-term monitoring conclusion that S20 delivers optimal phosphorus removal efficiency. A comparison of treatment performance between artificial and actual aquaculture tailwater showed no significant differences, indicating that the use of artificially prepared aquaculture tailwater can, to some extent, reflect the system’s effectiveness in real-world applications. This study revealed a dual mechanism of salinity effects: S20 enhances phosphorus immobilization by optimizing media surface charge properties and stimulating functional microbial activity, whereas S30 triggers structural risks in treatment media through iron over-leaching. Our findings demonstrate that S20 represents the optimal balance for high-efficiency phosphorus removal in IC-CWs treating actual aquaculture wastewater, with the system exhibiting strong adaptability to phosphorus loading fluctuations. This provides critical design parameters for full-scale implementation.

### 3.2. Iron Release Dynamics in IC-CWs Under Different Salinity Conditions

**Figure 5:**
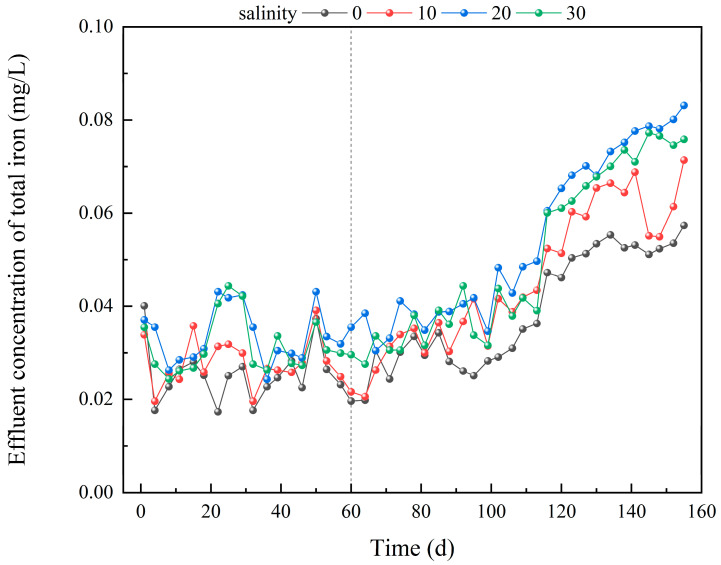
Precipitation of TFe in IC-CWs effluent at different salinities.

It has been reported that the iron content in water bodies directly influences the phosphorus removal efficiency of IC-CWs via chemical adsorption and precipitation reactions [26,27]. The primary mechanism involves the release of Fe^2+^/Fe^3+^ from IC materials during corrosion, which combine with dissolved phosphate to form stable iron-phosphate precipitates (e.g., FePO_4_, Fe_3_(PO_4_)_2_) [28,29]. In this study, effluent iron levels varied due to operational duration and salinity-dependent corrosion of iron-carbon substrates (Figure 5). During initial operation (1–60 days), TFe release levels in all systems remained below 0.05 mg L^−1^. This may be attributed to the incomplete establishment of the corrosion activation process in the iron-carbon substrates. In later phases (60–155 days). TFe release levels exhibited a gradient pattern: S20 (0.054 ± 0.018 mg L^−1^) > S30 (0.050 ± 0.018 mg L^−1^) > S10 (0.045 ± 0.015 mg L^−1^) > S0 (0.039 ± 0.012 mg L^−1^). Although iron release increased under S20 conditions, effluent total Fe concentrations remained below 0.1 mg L^−1^—well within the WHO guideline of 0.3 mg L^−1^ for aquatic environments—indicating no risk of secondary iron pollution. However, no significant difference was observed between the S20 and S30 groups (*p* > 0.05), which may be attributed to the inhibitory effect of high salinity (>20) on corrosion reactions [30,31]. The sustained iron release at S20 elevated TP removal by 5.90–24.30%, supplying essential reactants for chemical precipitation, including three key pathways [32,33]:

(1) Ferrous ion complex precipitation:

(1) $${\mathrm{Fe}}^{2+}\;+\;\mathrm{HP}O_{4}^{2-}\;\to \;\mathrm{FeHP}O_{4}↓$$

(2) $$3Fe^{2+}+2PO_{4}^{3-}\to Fe_{3}(PO_{4}{)}_{2}↓$$

(2) Iron oxidation precipitation:

(3) $$4Fe^{2+}\;+\;O_{2\;}+\;2H_{2}O\;\to \;4Fe^{3+}\;+\;4OH^{-}$$

(4) $$Fe^{3+}+PO_{4}^{3-}\to \;\mathrm{FeP}O_{4}↓$$

(3) Hydroxy iron oxide adsorption and coprecipitation:

(5) $$Fe^{3+}\;+\;3H_{2}O\;\to \;\mathrm{Fe}(\mathrm{OH}{)}_{3}↓\;+\;3H^{+}$$

**Figure 6:**
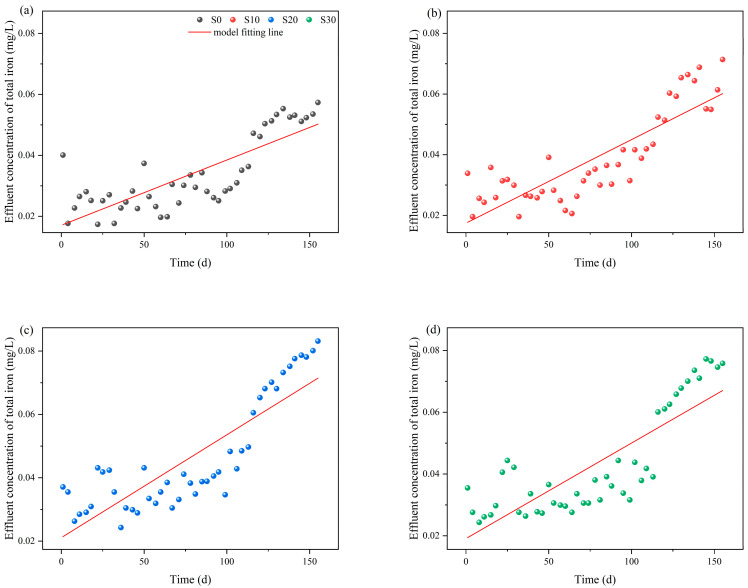
Linear fitted curves for TFe release rates based on salinity-time coupling, (**a**) S0; (**b**) S10; (**c**) S20; (**d**) S30.

When evaluating the dynamic iron release characteristics under varying salinity conditions, regression modeling revealed linear TFe accumulation with operational duration (Figure 6).

**Figure 7:**
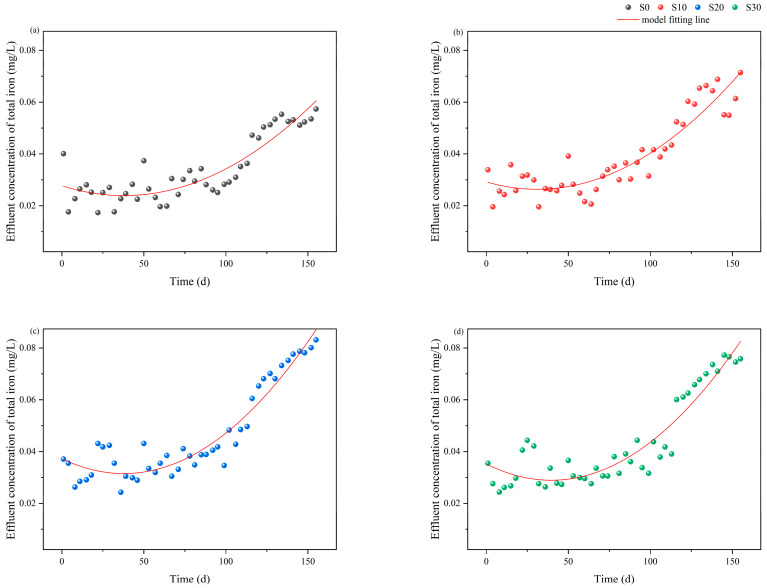
Polynomial fitted curves for TFe release rates based on salinity-time coupling. (**a**) S0; (**b**) S10; (**c**) S20; (**d**) S30.

The fitting results demonstrated that TFe concentration in the effluent exhibited a significant temporal increase (R^2^ = 0.65–0.72). In the linear regression model (C = bt + a), the slope parameter b represented the iron release rate, with the order of magnitude ranked as S20 > S30 > S10 > S0. Significant differences (*p* < 0.05) were observed between S0 and S20/S30, indicating that salinity levels ≥20 significantly enhanced the iron release rate and accelerated the corrosion process. Quadratic modeling (C = B_2_t^2^ + B_1_t + a; Figure 7) further improved the fit (ΔR^2^ = 0.14–0.19) and revealed two distinct kinetic shifts (Table 1). First, B_1_ exhibited negative values (−1.83 × 10^−4^ to −3.19 × 10^−4^), suggesting a decay in linear driving forces such as iron-carbon micro-electrolysis. Second, B_2_ showed significantly positive values (2.67 × 10^−4^ to 4.05 × 10^−4^), reflecting the increasing dominance of nonlinear processes, including precipitate desorption, microbially mediated iron transformation, and plant root-driven oxygen gradients. Notably, maximal B_2_ values at S20 and S30 (4.05 × 10^−4^ and 4.03 × 10^−4^, *p* < 0.01) confirmed that high salinity disrupted passivation layers and promoted non-steady-state iron release.

### 3.3. Microbial Analysis

#### 3.3.1. Analysis of Microbial Enzyme Activity in Wetland Substrate

**Figure 8:**
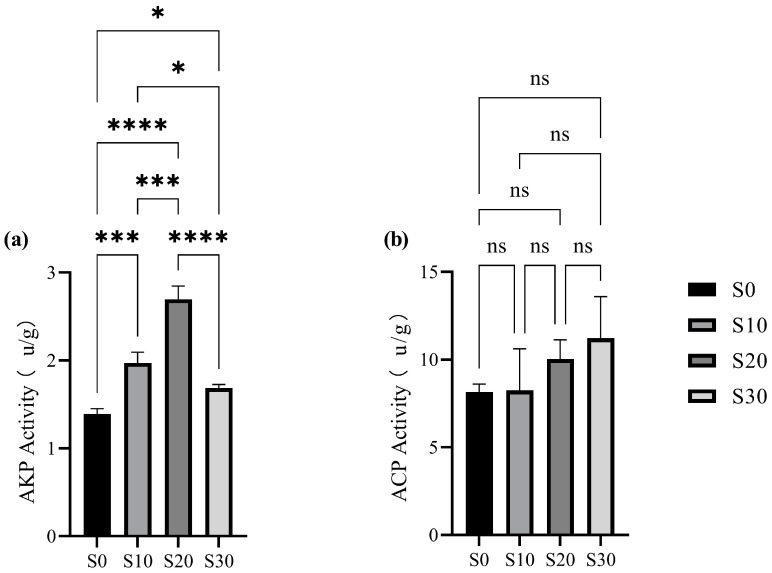
Changes in enzyme activities of AKP (**a**) and ACP (**b**) in different salinity systems. Note: The asterisk represents a statistically significant difference, and ns represents no statistically significant difference. * at *p* < 0.05, *** at *p* < 0.001, and **** at *p* < 0.0001.

Enzyme activity in PAOs critically influences microbial phosphorus removal efficiency [34]. Salinity modulates enzymatic catalysis by altering ionic strength and substrate binding [35]. We thus analyzed key enzyme activities governing phosphorus removal in IC-CWs under salinity stress (Figure 8).

AKP, a class of hydrolases ubiquitously present in organisms, plays a pivotal role in organic phosphorus mineralization by specifically hydrolyzing phosphate monoester bonds to release soluble PO_4_^3−^, thereby supplying the substrate for subsequent biological phosphorus removal processes [36]. AKP activity differed significantly across salinity groups (*p* < 0.05), ranking: S20 > S10 > S30 > S0. This trend closely aligned with the salinity response characteristics of phosphorus removal efficiency observed in long-term system monitoring. Notably, under a salinity of 20, the peak AKP activity demonstrated a significant correlation with the optimal TP removal efficiency. This phenomenon may be attributed to the dual mechanisms by which S20 enhances the stability of enzyme-substrate complexes:

(1) Ionic regulation of protein conformation: Optimal ion concentrations maintain the structural integrity of the AKP protein conformation, ensuring enzymatic functionality;
(2) Charge shielding effects: Saline ions reduce electrostatic repulsion between the enzyme and substrates through charge shielding, thereby improving the binding efficiency of organic phosphorus compounds.

At S30, AKP activity declined significantly versus S20 (37.40% reduction), likely due to enzyme denaturation [37]. The low activity at S0 suggests insufficient ionic strength impairs substrate binding despite absent salting-out effects.

ACP, another critical class of phosphatases, effectively hydrolyzes organic phosphorus compounds and participates in polyphosphate metabolism [38]. ACP is primarily secreted by wetland plants [39]. In contrast to AKP, ACP maintained high catalytic activity at elevated salinities (S20–S30), aligning with its reported salinity tolerance [40]. Key drivers include: (1) osmotic regulation, where elevated salinity stimulates plant ACP secretion [41]; and (2) iron-mediated catalysis, whereby salinity modulates iron valence shifts under anaerobic conditions, enhancing ACP-driven organic phosphorus mineralization [42]. This salinity-iron valence-ACP activity cascade suggests that IC-CWs can optimize iron precipitation via salinity control to boost phosphatase efficiency.

#### 3.3.2. Changes in Phosphorus-Accumulating Microbial Communities

**Figure 9:**
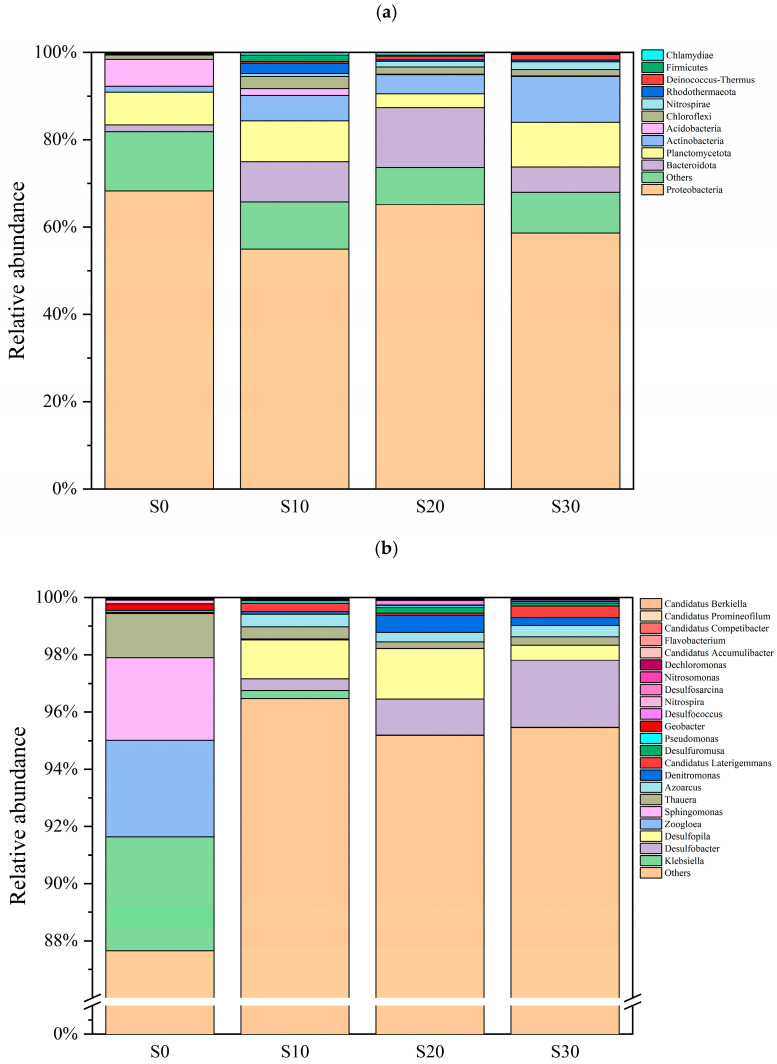
Changes in microbial abundance at the phylum level (**a**) and genus level (**b**) for each salinity system.

To further investigate the effects of varying salinity levels on the phosphorus removal capabilities of functional microorganisms, we analyzed microbial communities at the phylum and genus levels across four constructed wetland systems with distinct salinity conditions (Figure 9). The data revealed that dominant phyla in the four salinity-level wetland systems included *Proteobacteria*, *Actinobacteria*, *Bacteroidota*, *Firmicutes*, *Chloroflexi*, *Nitrospirae*, and *Planctomycetota*, which likely play critical roles in phosphorus removal processes.

**Figure 10:**
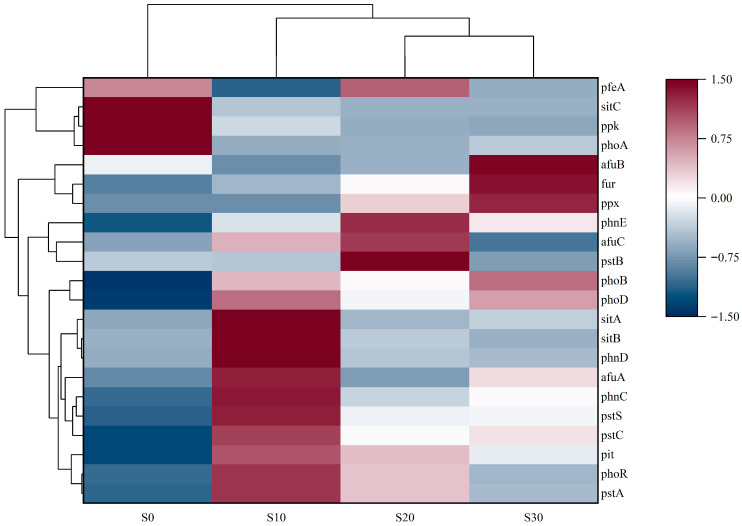
Abundance of phosphorus removal related genes expressed in each salinity system.

Among these, *Proteobacteria*, as the dominant phylum across all groups, exhibited a peak relative abundance of 68.25% in the S0 group, indicating its predominant role in the wetland substrate [43]. This phylum encompasses numerous phosphorus-accumulating bacteria capable of assimilating environmental phosphorus and storing it as polyphosphate. *Actinobacteria* significantly increased in the S30 group, with certain strains demonstrating the ability to degrade organic phosphorus via phosphatase secretion. The distribution of *Bacteroidota* showed significant variations across groups: S0 (1.54 ± 0.64%), S10 (9.23 ± 3.92%), S20 (15.40 ± 1.2%), and S30 (5.80 ± 3.40%). It is noteworthy that the specific enrichment of this phylum in the S20 group (a 10-fold increase compared to the S0 group) exhibited a positive correlation with the significantly elevated abundance of the *phnE* operon it harbored (Figure 10). The phosphate-specific ABC transporter system encoded by this gene cluster directly mediates the uptake and mineralization of environmental organic phosphorus compounds [44]. This mechanism likely accounts for the peak organic phosphorus removal efficiency observed under S20 salinity conditions. However, the decline in *Bacteroidota* abundance in the S30 group (9.6% reduction compared to S20) implies that high-salinity stress may suppress its metabolic activity or gene expression, thereby compromising phosphorus cycling functionality [45].

The relative abundance of the *Chloroflexi* phylum exhibited significant variations under different salinity gradients (*p* < 0.05), with specific values as follows: S0 group (0.98 ± 0.32%), S10 group (2.88 ± 1.21%), S20 group (1.62 ± 0.35%), and S30 group (1.42 ± 0.36%). Notably, the abundance in the S10 group was significantly higher than in other experimental groups. It is noteworthy that the abundance of *Chloroflexi* in saline systems (S10, S20, S30) was significantly elevated compared to the non-saline control group (S0). This observation aligns with the findings of Wang et al. [46], who reported the salt-tolerant characteristics inherent to this phylum. Further analysis revealed that the *Chloroflexi* community may play a pivotal role in phosphorus removal processes within IC-CWs, with its contribution level demonstrating a positive correlation to the salinity gradient (S10 > S20 > S30). Kragelund et al. [47] have confirmed the pivotal role of this phylum in biological phosphorus removal processes. Our results further demonstrate that optimal salinity conditions (salinity 10) can enhance the phosphorus removal efficiency of the system by promoting *Chloroflexi* growth. However, when salinity exceeds a critical threshold (salinity 30), this promoting effect diminishes significantly. Compared to the non-saline control group (S0: 1.37 ± 0.46%), the relative abundance of *Actinobacteria* in the S10, S20, and S30 groups increased significantly by 4.23-fold (5.80 ± 0.25%), 3.26-fold (4.46 ± 1.05%), and 7.69-fold (10.54 ± 7.92%), respectively. While the S30 group exhibited the greatest enhancement, its higher data dispersion (RSD = 75%) suggests that salinity exceeding the threshold may induce divergent responses in microbial community dynamics. Numerous studies have identified this bacterium as a pivotal phosphate-solubilizing bacterium (PSB) in phosphate immobilization, further corroborating the regulatory role of salinity gradients in modulating its abundance and phosphorus metabolic activity [48,49].

At the genus level, distinct microbial taxa dominate phosphorus removal functions across systems with varying salinity regimes. Under zero-salinity conditions, non-salt-tolerant phosphorus-accumulating bacteria—including *Klebsiella*, *Zoogloea*, and *Sphingomonas*—were predominant in the wetland system, accounting for 3.99%, 3.38%, and 2.88% of the microbial community, respectively. The phosphorus removal process is mediated through the canonical biological phosphorus accumulation pathway, which aligns with the microbial community traits commonly observed in freshwater CWs [50]. As salinity increased, the microbial community within the system underwent marked shifts. The abundance of salt-tolerant phosphorus-accumulating taxa, such as *Desulfopila* (0.53–1.75%) and *Desulfobacter* (0.41–2.34%), rose correspondingly, establishing these genera as dominant populations in high-salinity environments. Both microorganisms, classified as sulfate-reducing bacteria (SRB), are enriched under high-salinity conditions. They likely enhance ferric phosphate precipitation via sulfide-ferric ion interactions, thereby partially offsetting losses in biological phosphorus removal [51]. *Candidatus Accumulibacter*, a prototypical phosphorus-accumulating bacterium, plays a critical role in biological phosphorus removal processes. The data revealed that the abundance of *Candidatus Accumulibacter* across all salinity gradients (S0–S30) remained extremely low (0.01–0.02%), suggesting that the activity of PAOs in IC-CWs may be constrained or their phosphorus removal function partially supplanted by alternative mechanisms, such as chemical precipitation. Furthermore, the iron-reducing bacterium *Geobacter* exhibited higher activity under low-salinity conditions (S0: 0.23%) but was inhibited in high-salinity environments, potentially impairing the formation of ferric phosphate. The substantial proportion of unclassified microorganisms (87.65–96.48%) underscores the current limitations of microbial databases in characterizing functional strains within CWs. Metagenomic analysis to elucidate the metabolic potential of this unexplored microbial cohort may provide critical insights into novel salt-tolerant phosphorus removal mechanisms, offering a breakthrough avenue for optimizing CWs performance under saline conditions. The aforementioned findings demonstrate that salinity stress drives the transition of constructed wetland systems from a biologically dominated phosphorus accumulation regime to a chemically enhanced phosphorus removal regime through dual regulatory mechanisms—functional microbial community restructuring and metabolic pathway shifts.

#### 3.3.3. Variations in Functional Gene Abundance of PAOs

Analysis of functional gene abundance of PAOs under different salinity levels revealed distinct patterns (Figure 10). At S0, the majority of PAOs’ functional genes (*pstC*, *pstS*, *phnD*) exhibited significantly lower abundance, indicating minimal expression of phosphorus removal-associated genes in non-saline environments. At S10, the abundance of phosphate transporter-related genes (*pstC*, *pstS*) and organic phosphorus mineralization genes (*phnD*) was significantly upregulated. Notably, pstS expression increased 14.25-fold relative to S0, indicating that salt stress markedly activated the high-affinity phosphate transport system in PAOs. This regulatory mechanism of gene expression is likely associated with cellular osmoregulation, whereby PAOs enhance phosphate uptake to maintain intracellular phosphorus homeostasis and provide substrates for the synthesis of compatible solutes, such as polyphosphate [52]. However, at higher salinities (S20, S30), the expression levels of the *pstS* gene declined to 6.70-fold and 6.83-fold of the S0 group, respectively, demonstrating a highly significant salinity-induced suppression compared to S10 (*p* < 0.01). This attenuation in expression magnitude unveils the dual role of salinity stress: S10 stimulates phosphorus metabolic activity in PAOs, while supra-threshold salinity (salinity > 10) reduces the efficacy of the transport system. It is hypothesized that excessively high salt concentrations may compromise transmembrane proton gradients or elicit ion-specific toxicity, thereby destabilizing the conformational stability and functional expression of transport proteins [53]. This discovery provides critical molecular evidence for elucidating the salinity response threshold of PAOs in IC-CWs.

At S20, the system exhibited differential gene responses characteristics, marked by a decline in the abundance of certain genes (*ppk*, *phoA*). However, genes such as *pstC, pstS, phoD*, and *ppx* retained elevated expression levels, suggesting their relative insensitivity to salinity stress and continued functional importance under high-salinity conditions. Notably, the abundance of the *ppx* gene, associated with polyphosphate (*poly-P*) degradation, significantly increased at S20 (*p* < 0.01), suggesting its potential role in degrading polyphosphate under salinity stress to provide energy or regulate intracellular osmotic pressure [54].

At S30, the phosphorus metabolic gene network undergoes significant restructuring. While the abundance of certain functional genes declines markedly (*ppk*, *phoA*), others exhibit a substantial increase (*ppx*, *phoB*, *phoD*), suggesting that these upregulated genes may participate in specific metabolic adaptation or stress response mechanisms under hypersaline conditions. Concurrently, the abundance of the *ppx* gene progressively increased with escalating salinity levels, further suggesting its potential role in aiding cellular adaptation to high-salinity stress via polyphosphate degradation. This systematic shift in gene expression patterns indicates that when salinity exceeds the physiological tolerance threshold of PAOs, cells may activate *poly-P* catabolic pathways to acquire energy for maintaining osmotic equilibrium, rather than relying on energy-intensive active transport systems for phosphorus uptake [55]. Additionally, *sitA* maintained elevated expression levels under salinity 10 but exhibited a significant decline in abundance under higher salinity conditions. This phenomenon may be attributed to the role of *sitA* in mediating iron ion transport under S10. In contrast, under hypersaline environments, its function may be either compensated by alternative transport systems or downregulated at the transcriptional level, thereby diminishing its contribution [56]. This phenomenon may be linked to functional compensation of siderophore systems or ion competition effects under hypersaline conditions. These findings demonstrate that the functional gene expression levels of PAOs are closely correlated with salinity variations. However, changes in gene abundance do not linearly track salinity shifts but instead reflect adaptive responses to enable cellular acclimatization to differing salinity stress regimes.

## 4. Conclusions

This study demonstrates that IC-CWs effectively remove phosphorus from saline aquaculture tailwater through synergistic physicochemical and microbial processes, with optimal total phosphorus removal (78.80 ± 6.01%) achieved at salinity 20 through enhanced ferric phosphate precipitation (52% increased iron release) and upregulated P-transport genes (*pstS*, *phnE*) in PAOs. While salinity ≤ 20 promoted microbial P metabolism by enriching *Bacteroidota* and *Chloroflexi* (facilitating organic P mineralization), excessive salinity (30) suppressed PAO activity (34% *AKP* reduction) and altered iron dissolution kinetics (R^2^ = 0.88–0.91), revealing a threshold for microbial functionality and highlighting the need for periodic substrate regeneration due to passivation layer rupture under hypersaline conditions. Although conducted under laboratory-scale and short-term conditions, this study contributes to building a conceptual basis for salinity optimization in IC-CWs treating saline aquaculture tailwater. From an engineering perspective, maintaining a salinity level of approximately 20 may improve phosphorus removal efficiency but could also influence operational complexity and cost. In practical aquaculture systems, salinity regulation requires additional salt input and energy consumption, which might increase overall maintenance expenses. Therefore, future studies should further evaluate the techno-economic feasibility of maintaining optimal salinity conditions under real-scale IC-CW operations.

## Figures and Tables

**Table 1:** Comparison of TFe release kinetic model parameters under different salinity conditions.

Salinity Groups	Slope of the Linear Model (b)	Linear Term Coefficient (B_1_)	Quadratic Term Coefficient (B_2_)	R1^2^	R2^2^	ΔR^2^
S0	2.14 × 10^−4 a^	−2.02 × 10^−4 a^	2.67 × 10^−4 a^	0.65	0.81	0.17
S10	2.76 × 10^−4 ab^	−1.83 × 10^−4 ab^	2.94 × 10^−4 ab^	0.72	0.85	0.14
S20	3.25 × 10^−4 b^	−3.06 × 10^−4 b^	4.05 × 10^−4 b^	0.72	0.91	0.19
S30	3.09 × 10^−4 b^	−3.19 × 10^−4 b^	4.03 × 10^−4 b^	0.68	0.88	0.19

Note: Different superscript letters denote significant differences in TFe release among salinity groups (one-way ANOVA); ΔR^2^ = R2^2^ (polynomial model) − R1^2^ (linear model).

## Data Availability

The data that support the findings of this study are available from the corresponding author upon reasonable request.
